# Aortic haemodynamics and wall stress analysis following arch aneurysm repair using a single-branched endograft

**DOI:** 10.3389/fcvm.2023.1125110

**Published:** 2023-05-22

**Authors:** Sampad Sengupta, Xun Yuan, Ludovica Maga, Selene Pirola, Christoph A. Nienaber, Xiao Yun Xu

**Affiliations:** ^1^ Department of Chemical Engineering, Imperial College London, London, United Kingdom; ^2^ National Heart and Lung Institute, Faculty of Medicine, Imperial College London, London, United Kingdom; ^3^ Cardiology and Aortic Centre, Royal Brompton and Harefield Hospitals, Guy’s and St Thomas’ NHS Foundation Trust, London, United Kingdom; ^4^ Department of Electronics, Information and Bioengineering, Politecnico di Milano, Milan, Italy; ^5^ Department of Biomechanical Engineering, Delft University of Technology, Delft, Netherlands

**Keywords:** TEVAR, endograft, aortic arch, computational fluid dynamics, finite element analysis

## Abstract

**Introduction:**

Thoracic endovascular aortic repair (TEVAR) of the arch is challenging given its complex geometry and the involvement of supra-aortic arteries. Different branched endografts have been designed for use in this region, but their haemodynamic performance and the risk for post-intervention complications are not yet clear. This study aims to examine aortic haemodynamics and biomechanical conditions following TVAR treatment of an aortic arch aneurysm with a two-component single-branched endograft.

**Methods:**

Computational fluid dynamics and finite element analysis were applied to a patient-specific case at different stages: pre-intervention, post-intervention and follow-up. Physiologically accurate boundary conditions were used based on available clinical information.

**Results:**

Computational results obtained from the post-intervention model confirmed technical success of the procedure in restoring normal flow to the arch. Simulations of the follow-up model, where boundary conditions were modified to reflect change in supra-aortic vessel perfusion observed on the follow-up scan, predicted normal flow patterns but high levels of wall stress (up to 1.3M MPa) and increased displacement forces in regions at risk of compromising device stability. This might have contributed to the suspected endoleaks or device migration identified at the final follow up.

**Discussion:**

Our study demonstrated that detailed haemodynamic and biomechanical analysis can help identify possible causes for post-TEVAR complications in a patient-specific setting. Further refinement and validation of the computational workflow will allow personalised assessment to aid in surgical planning and clinical decision making.

## 1. Introduction

An aortic aneurysm is a localised distention of the vessel wall, resulting in an abnormal and often permanent dilatation of the affected section of the aorta. Thoracic aortic aneurysms (TAAs) can arise in the ascending aorta, aortic arch, thoracic descending aorta, or the thoraco-abdominal regions of the aorta. Isolated arch aneurysms are less frequent but pose a significant challenge given the geometric complexity of the region, especially the involvement of supra-aortic vessels that are responsible for supplying blood to the head and upper parts of the body. Insufficient blood perfusion to the arch branches can result in severe and often fatal consequences (1–3). The standard treatment option for arch aneurysm is open-chest surgery, with thoracic endovascular aortic repair (TEVAR) providing a minimally invasive alternative. Initially introduced for the treatment of abdominal aortic aneurysms, endovascular repair has now been extended to the thoracic aorta and arch. TEVAR offers several benefits to patients, including short post-operative time spent in the hospital and fast recovery (4–6).

Endografts used for TEVAR are designed to mimic a patient's anatomy as closely as possible. They can be branched or unbranched, depending on the zone of the aorta in which they are deployed. Improvements have been made to the design and deployment methods of endografts, yielding a remarkable decrease in mortality and morbidity rates of the repair procedure (4, 7). Branched or fenestrated stent-grafts are often used to ensure perfusion of blood to the supra-aortic vessels when implanted in the arch. Branched stent-grafts are conceptually more appealing than fenestrated devices as they are adaptable to a wide range of anatomical morphologies. Branched stent-grafts can be manufactured as either single- or multi- branched endografts with or without inner tunnels. These inner tunnels can be either antegrade or retrograde with antegrade tunnel branches tending to provide a smoother transition of flow into the arch branches as reported in previous computational studies (8). Single-branched stent-graft requires two bypass connections between the upper branches, e.g., bypass between the innominate artery and the left common carotid artery or between the left subclavian artery and the left common carotid, and thus may result in insufficient blood perfusion to the supra-aortic arteries as the entire flow is supplied by a single bridging stent. Double-branched endografts are developed for zone 0 deployment with two bridging stents connected to the innominate and left common carotid arteries (9, 10). The choice of endograft used lies with clinicians and is based on the treatment procedure, deployment zone, and other peri-operative factors.

Implanting an endovascular device will obviously change the flow within the repaired region, and it is of particular interest to gain more insights into the haemodynamic changes induced by the endograft (11, 12). *In vivo* examinations and clinical imaging alone cannot provide information on certain parameters of interest such as wall shear stress (WSS), forces exerted on the wall, and localised flow patterns. Previous studies have examined the performance of endografts in the aortic arch and their ability to perfuse the arch branches. For example, Zhu et al. (13) and Sengupta et al. (14) performed computational fluid dynamics (CFD) analysis of aortic flow after implantation of double-branched endografts in patients with arch aneurysms and noted improved flow patterns with increased WSS in the aortic arch. However, the presence of such inner branches can lead to disturbed flow in the region. In addition, arch endografts often involve occlusion of the native ostia of supra-aortic branches and peri-operative revascularisation procedures are required to maintain perfusion to the arch branches (15). Left subclavian artery revascularisation, often through the left common carotid artery, can lead to increased flow in the remaining native vessels with increased peak flow velocity and higher displacement forces being exerted in the region (16, 17).

**Figure 1:**
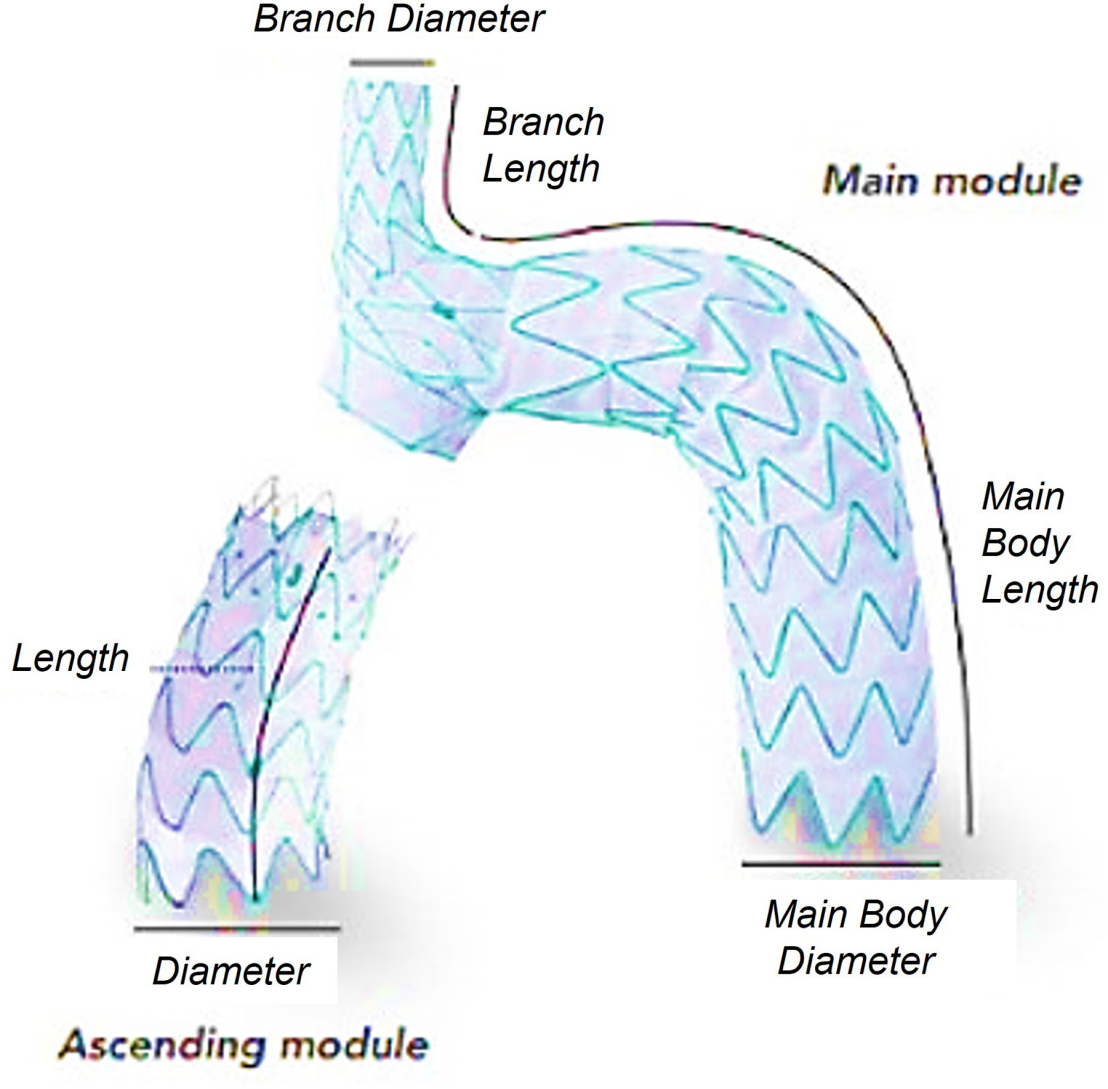
The NEXUS™ device selected for TEVAR, highlighting its features and different components.

The influence of endografts on flow in the repaired aorta depends strongly on their design, and here the focus is on the effect of a single-branched device with no inner tunnel branches, suitable for zone 0 deployment to treat aortic arch pathologies. The device implanted in the patient included in this investigation is the Nexus™ Aortic Arch Stent Graft System developed by Endospan (Herzlia, Israel). As shown in Figure 1, it consists of a main module for the aortic arch and descending aorta with a side-branch for one supra-aortic vessel and a curved module for the ascending aorta that connects to the main module through a self-protecting sleeve (18). The device is suitable for implantation in zone 0 to zone 2 in the thoracic aorta and the branch emerging from the main module serves as an anchoring mechanism to hold the component in place. Being an “off the shelf” device, the length of each module and the diameters are chosen based on the application and anatomical features of the aorta being treated (19). The proximal end of the device has curved stent tips designed for atraumatic sealing in the ascending aorta and is meant to reduce pressure points on the outer curvature. The two modules overlap and have a radial force interlocking mechanism holding the separate components together in the ascending region.

This investigation aimed to examine the haemodynamic and biomechanical conditions of the aorta following TEVAR treatment for an aortic arch aneurysm with the Nexus™ device. We used patient-specific geometric models reconstructed from computed tomography (CT) scans acquired before and after the interventional procedure and at follow-up.

## 2. Materials and methods

### 2.1. Patient data

**Figure 2:**
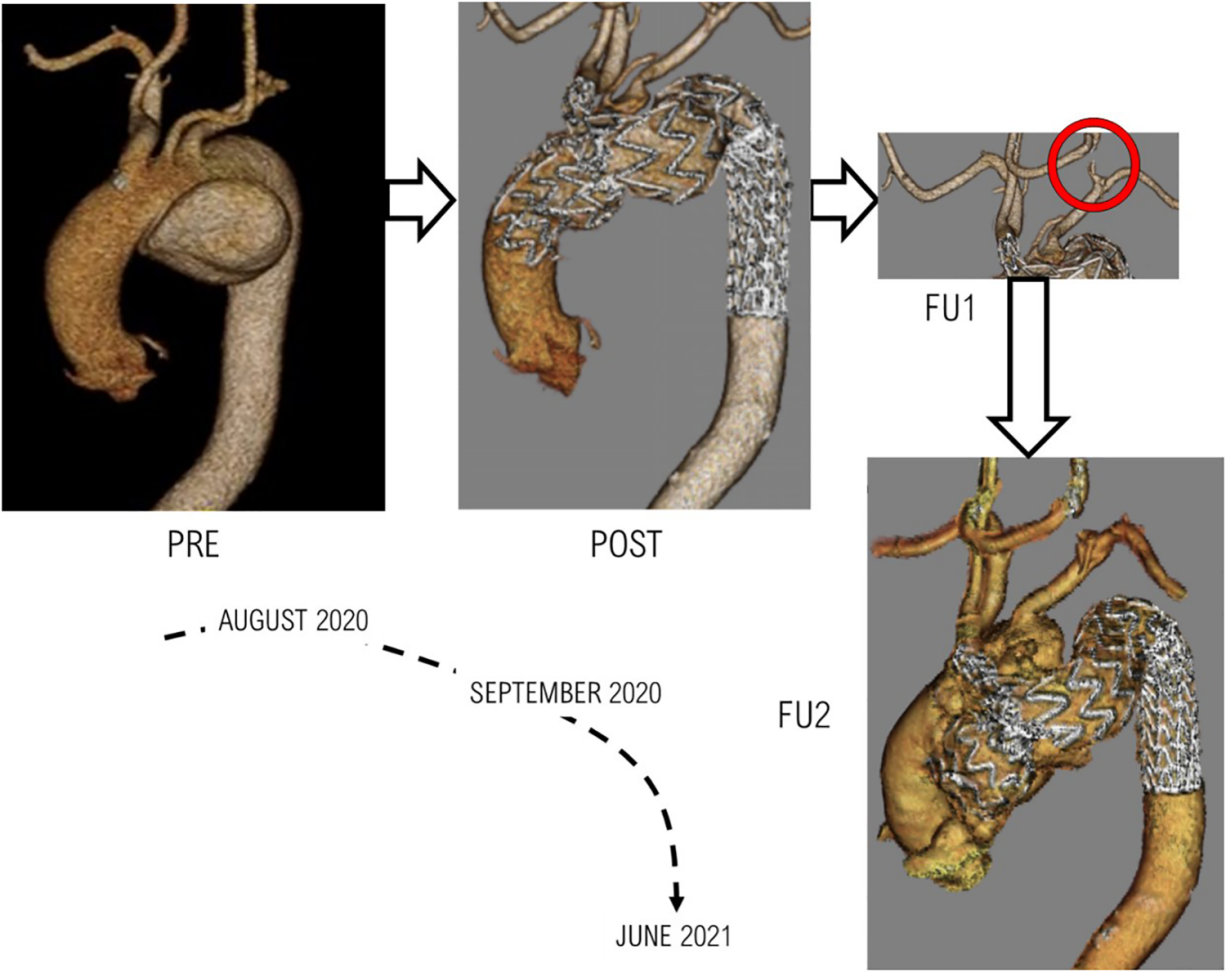
The various stages of treatment and follow-ups for the patient examined. PRE: Pre-TEVAR geometry indicating presence of aneurysm, POST: Post-TEVAR geometry following a successfully completed procedure, FU1: First follow-up scan which indicated negative results in the form of the carotid-carotid bypass being compromised, FU2: The final clinical images obtained from the patient indicating the formation of an aneurysmal sac in the ascending aorta.

Clinical data and images were acquired from a patient with a large aneurysm arising from the inferior wall of the aortic arch. The patient underwent TEVAR with a single-branched Nexus™ device. Figure 2 outlines the timeline of the case being studied, with reconstructions from clinical images showing the progression of the case. Ethical approval was obtained from the local Ethics Committee, and written consent was given by the patient.

**Figure 3:**
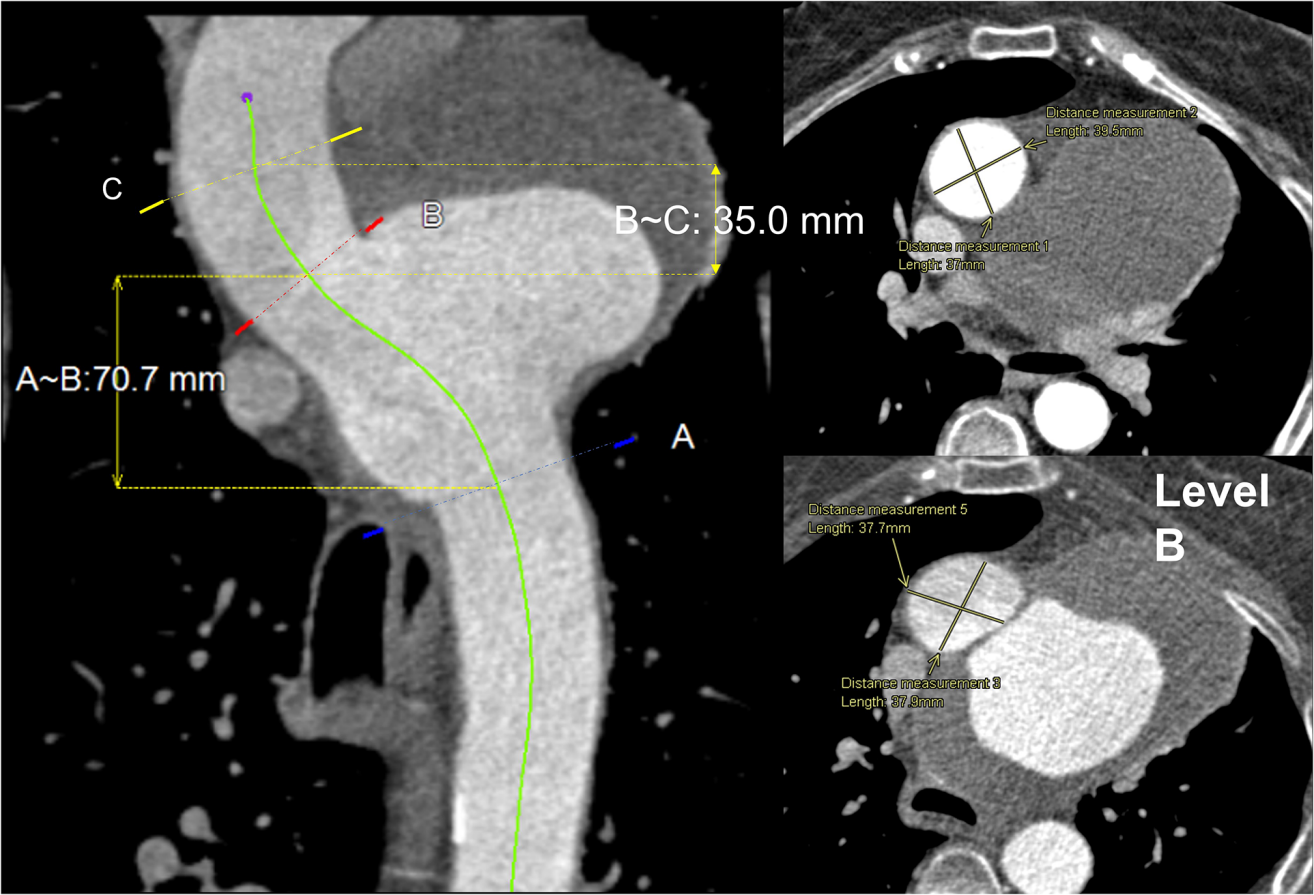
Central line view of the whole aorta: (left) proximal to distal aorta with three marked levels. (**A**)—just beyond LSA, (**B**)—just before IA, (**C**)—proximal landing zone, 35** **mm from IA proximally to the ascending aorta. The length between (**B**) and (**C**) is 35** **mm, and (**B**) and (**A**) is 70.7** **mm. (Right) Transverse view of levels (**B**) and (**C**) with a cross-sectional measurement of aortic diameter 37.9 **× **37.7** **mm and 37.0 **× **39.5** **mm, respectively.

The aneurysm, measuring 9.6 × 6.0 × 5.9 cm in the aneurysmatic sac, led to compressions in the distal pulmonary vessels but there were no morphometric changes to the innominate artery (IA), left common carotid (LCC) artery and left subclavian artery (LSA). Pre-intervention CT images with measurements of diameters and distances at three different locations are shown in Figure 3.

The patient was treated with TEVAR using the Nexus™ device; the intervention was carried out following a debranching procedure to set up a bypass between the right common carotid (RCC) and LCC, as well as the LCC and LSA. The procedure was successful and restored flow in the arch whilst successfully excluding the aneurysm, with a suitable proximal sealing length of 3.5 cm from the IA, greater than the minimum recommended length of 3.0 cm. The measured landing diameter of the ascending aorta was 38 mm, and that of the device was 43 mm, thereby producing an oversize ratio of 13.2%. The aneurysmal sac was successfully sealed, both LCC-RCC bypass and LCC-LSA bypass were patent. However, follow-up scans indicated the LCC-RCC bypass remained patent whilst the LCC-LSA bypass was compromised with a thrombus in the bypass near the LCC vascular stroma. A final follow-up of the patient 10 months after the intervention revealed the formation of an aneurysmal sac in the ascending aorta with migration of the ascending module of the device and a suspected leak or tear in the ascending aorta.

The patient had other comorbidities which needed to be taken into consideration during the treatment planning stages. Prior to intervention, the patient was on alendronate 10 mg OD, atorvastatin 20 mg OD, folic acid 5 mg BD, methotrexate 20 mg weekly, omeprazole 20 mg OD, prednisolone 5 mg BD. Following the intervention, the medication was adjusted to alendronate 70 mg OD, aspirin 75 mg OD (for 3 months), atorvastatin 20 mg OD, bisoprolol 1.25 mg OD, clopidogrel 75 mg OD (for 3 months), folic acid 10 mg weekly, methotrexate 15 mg weekly, omeprazole 20 mg OD, prednisolone 10 mg OD.

### 2.2. Geometric models

**Figure 4:**
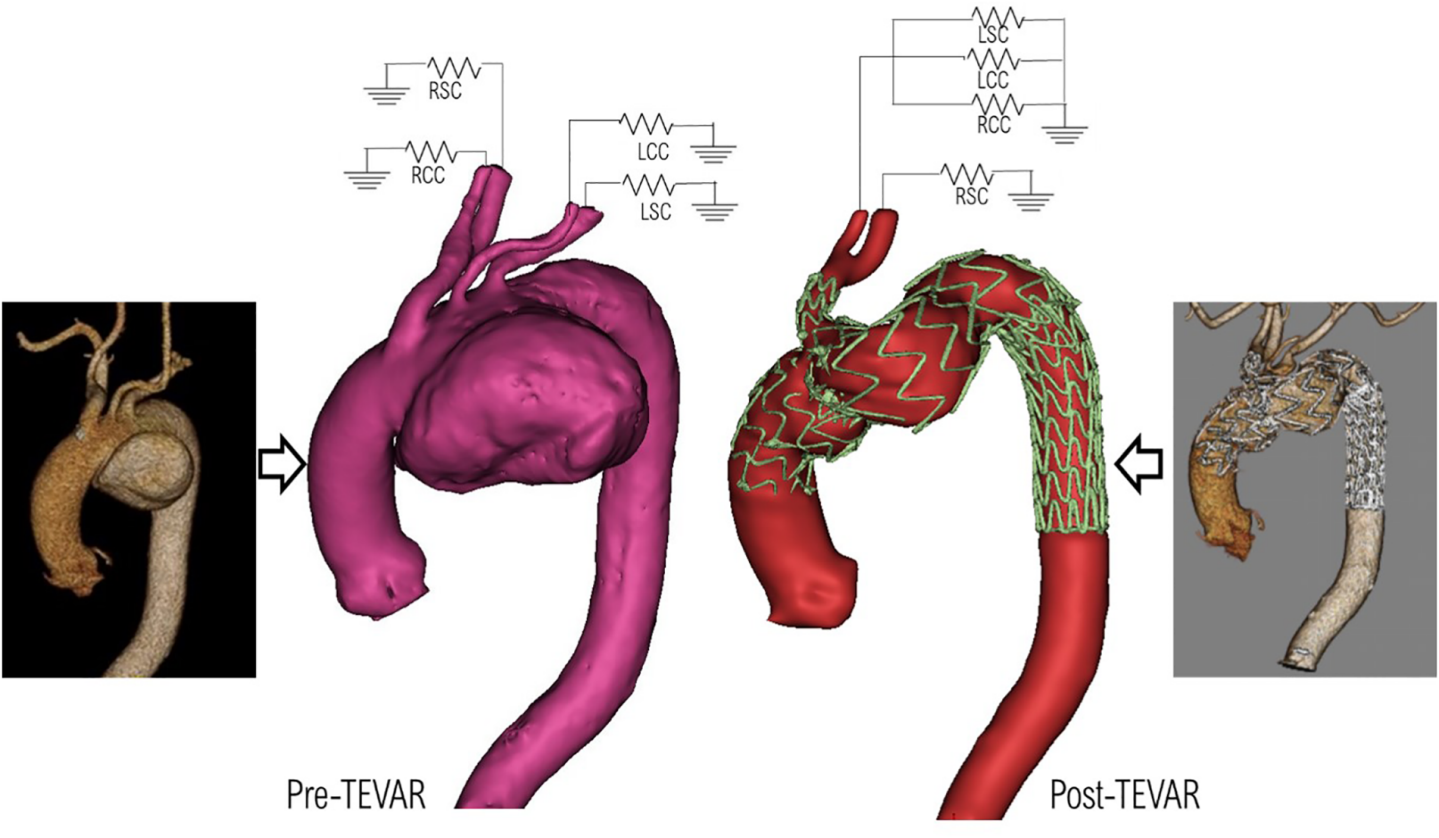
Pre-TEVAR **(left)** and post-TEVAR **(right)** models reconstructed from the corresponding CT scans, and the prescribed 3-EWM boundary conditions at the model outlets.

Anatomically accurate 3D geometries were reconstructed based on CT scans acquired using an ECG-gated spiral CT scanner (Siemens Somatom) at various stages as described in Figure 2. Image segmentation and 3D reconstruction of the aorta were performed using an image processing software, Mimics (v20, Materialise, Leuven, Belgium). A thresholding technique was adopted to isolate the regions of interest (ROI). User-defined lower and upper limits of grayscale intensity were set for thresholding, with a typical lower limit of 270–280 HU and an upper limit of 2,000 HU or above depending on the image resolution. This produced initial 2D masks on all the available slices. A split mask function was then used to separate the ROI from unwanted neighboring tissues that might have been included in the initial masks. The separated masks were manually inspected and modified if necessary to ensure all pixels in the targeted vessels were selected. Finally, the reconstructed geometries were smoothed using the Discrete Gaussian filter based on a linear smoothing enhancement algorithm. The smoothing function requires a smoothing factor (within range 0–1) and the number of iterations (within range 1–500) to be specified. A specific “compensate shrinkage” feature was enabled to preserve the shape of the geometry, thereby preventing the lumen of the aorta and its branches from shrinking. This was ensured by comparing vessel diameters extracted from the reconstructed 3D surface with those measured in the CT images at multiple locations of interest. Sensitivity tests indicated that setting a smoothing factor of 0.1 and 50 iterations for each stage of smoothing produced reliable reconstructions used in this study. Figure 4 shows the pre-TEVAR geometry which featured a large aortic arch aneurysm in the inferior radius of the arch and three supra-aortic branches emerging from the arch. The model also included the right brachiocephalic artery and RCC which bifurcate from the IA, resulting in four model outlets in the arch. The post-TEVAR geometry (Figure 3) incorporated the implanted device where the two modules were considered as one body and connected with the unstented portions of the aorta. The IA branch and its bifurcation were included while the LCC and LSA were excluded as they were covered by the main module. As a result, the post-TEVAR geometry had two outlets in the arch.

**Figure 5:**
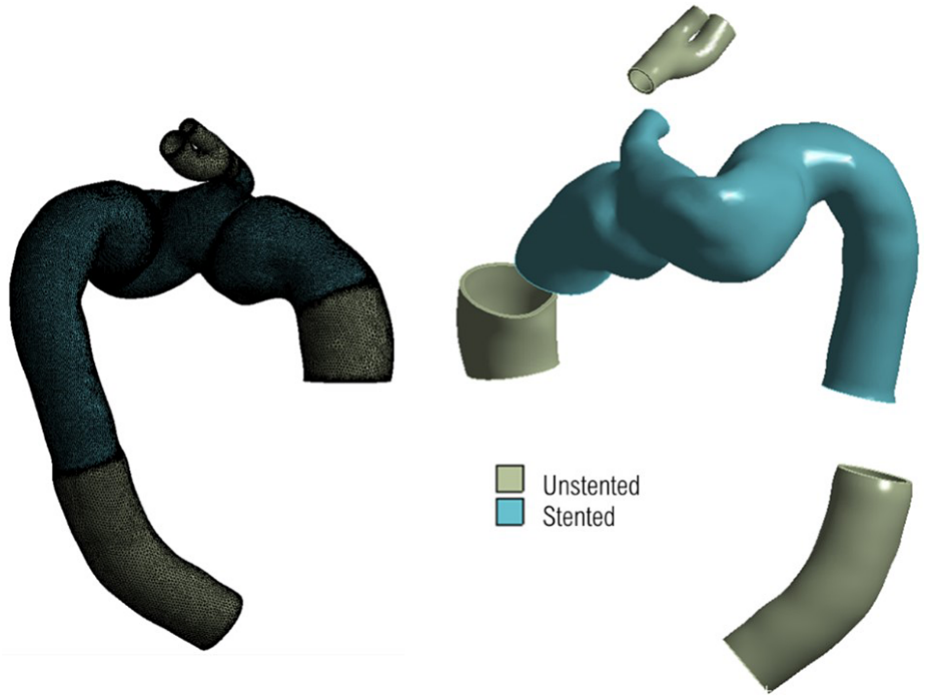
3d geometry for structural domain, with unstructured mesh shown on the **left**, and segments of different material highlighted on the **right**. The uniform thickness of the geometry can be seen clearly on the **right** with the branch inlets and outlets serving as regions of fixed points to tether the geometry in place.

Mesh generation was carried out using ANSYS ICEM CFD (v15.0, ANSYS Inc., Canonsburg, PA, USA). For haemodynamic analysis, unstructured meshes consisting of 10 prismatic boundary layers at the wall were generated with approximately 6.8 and 5 million elements in the post-TEVAR and pre-TEVAR geometries respectively. For biomechanical structural analysis, a constant wall thickness of 1.4 mm was applied to the reconstructed 3D luminal surface, creating a solid volume representing the aortic wall. Figure 5 shows the solid domain geometry consisting of 6.8 million elements and the delineation of the stented and unstented regions.

### 2.3. Computational details and boundary conditions

Flow in the aorta was described by the transient, three-dimensional equations for conservation of mass and momentum. Blood was modelled as a Newtonian fluid with a constant density of 1,060 kgm^−3^ and dynamic viscosity of 0.004 Pa s. Based on the measured peak flowrate of 2.34 × 10^−4 ^m^3^/s and inlet area of 993.13 mm^2^, the peak Reynolds number was 2,182.3 and the corresponding Womersley number was 19.2. This combination indicated that flow in the ascending aorta was likely to be disturbed (20), hence the need to employ the SST-Tran (shear stress transport—transitional) model, which has been shown to be more suitable for physiological flows involving potential transition from laminar to turbulent flow (21, 22).

**Figure 6:**
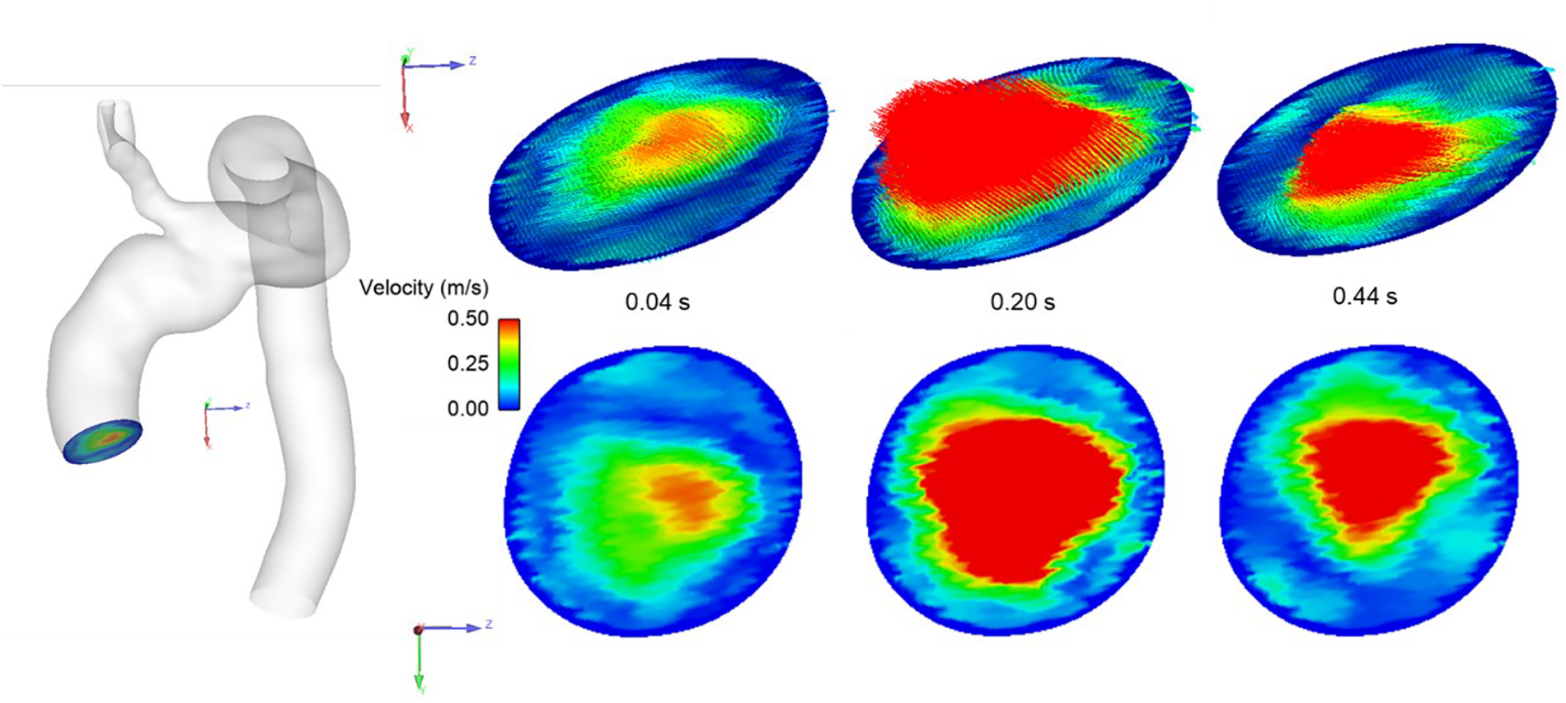
Representative 3D inlet velocity profile imposed at model inlet showing velocity distribution at different stages of the systolic phase of the cardiac cycle.

In order to solve the flow governing equations numerically, suitable boundary conditions at the inlet and outlets are required. These should, as much as possible, represent the flow conditions specific to the patient and the stage of treatment being considered. The inflow waveform was adapted from a previous study (22) and then adjusted to represent the recorded cardiac output of the patient and further tuned to the dimensions of the model inlet (23, 24). The lack of patient-specific inflow data, such as 4D Flow MRI specific to the patient, necessitated the implementation of a novel method for generating realistic 3D inlet velocity profiles (IVPs). A synthetic dataset of virtual aortic velocity profiles was generated by employing statistical shape modelling (SSM) to a clinical dataset consisting of 31 thoracic aortic aneurysm (TAA) cases; this produced representative 3D IVPs comparable to that of the velocity distributions observed whilst using specific 4D Flow MRI data (25). The velocity profile producing peak systolic velocity closest to that of the clinical measurement was chosen and interpolated in time to match the length of the cardiac cycle. Figure 6 shows the generated IVPs prescribed at the model inlet. The length of the cycle was adjusted according to the heart rate (HR) of the patient (Pre-TEVAR HR: 74 beats/minute, Post-TEVAR HR: 84 beats/min).

A 3-element Windkessel model (3-EWM) was prescribed at the outlets and tuned according to blood pressure measurements made throughout the observation and treatment phases (pre-TEVAR aortic pressure: 119/72 mmHg, post-TEVAR brachial pressure: 133/62 mmHg) using an established method (26). Since the 3-EWM requires mean arterial pressure (MAP) values for the necessary parameters to be set, MAP was calculated from the measured systolic and diastolic pressures (SP and DP respectively) (27).

$$MAP=\;DP+\frac{1}{3}(SP-DP)$$

Since the measured post-TEVAR pressure corresponded to the brachial (*brach*) pressure, which cannot be directly utilised for the 3-EWM, the measured brachial pressure was converted to central (*cent*) pressure using the following expression (28), with $DP_{cent}=DP_{brach}$,

$$SP_{brach}\;\approx \;0.83SP_{cent}+0.15DP_{cent}$$

All the model parameters used in the 3-EWM are given in Table 1. In addition, the employment of SST-Tran model required a turbulence intensity (Tu) to be prescribed at the inlet. A low Tu of 1% was set based on previous experience (21, 22). The wall was assumed to be rigid with a no-slip boundary condition. CFD simulations were carried out using ANSYS CFX v15.0 (ANSYS, Canonsburg, PA, United States) with a fixed time-step of 0.001 s and a convergence criterion of 10^−5^. All simulations were run for at least 3 cycles until a periodic solution was reached.

**Table 1:** 3-EWM parameters used in the three models simulated in this study.

Model	Outlet	R1 [Pa s m^−3^] (× 10^7^)	C [m^3^ Pa^−1^] (× 10^−9^)	R2 [Pa s m^−3^] (× 10^8^)
PRE	RSA	5.75	1.07	16.2
	RCCA	9.94	0.669	25.8
	LCCA	24.4	0.399	42.4
	LSA	9.63	0.868	19.7
	OUT	1.20	6.98	2.45
POST	RSA	5.75	1.03	16.8
	RCCA	4.02	1.03	9.19
	OUT	1.20	5.13	2.54
FU1	RSA	5.75	1.03	1.68
	RCCA	6.91	1.03	16.7
	OUT	1.20	7.56	2.25

RSA, Right subclavian artery; RCCA, Right common carotid artery; LCCA, Left common carotid artery; LSA, Left subclavian artery; OUT, model outlet).

For finite element analysis (FEA) of wall deformation and stress in the post-TEVAR model, the native aorta was assumed to be an isotropic, homogeneous and linear elastic material with a Young's modulus (E) of 0.8 MPa and Poisson's ratio (*ν*) of 0.49 (29). The stented region was also modelled as a linear elastic material with a Young's modulus of 15 MPa and Poisson's ratio of 0.3 (30, 31). The material densities were 1,100 kg.m^−3^ and 2,140 kg.m^−3^ for the native aorta and stented region respectively (30) (Molony et al., 2009). Since the aortic wall model was reconstructed from CT images obtained at diastole, it was necessary to account for prestress in the aorta under diastolic pressure conditions. Prestress was estimated using an iterative approach proposed and evaluated in previous studies (32, 33). The iterative process was carried out until the maximum total deformation in the stressed configuration was less than 0.5 mm under a diastolic pressure, allowing for the structural domain to achieve equilibrium with the internal blood pressure (34). The obtained prestress tensor was then applied in the FEA simulation where the geometry was tethered at the inlet and branch outlets and peak systolic pressure distribution from the flow simulations were applied as the load at the internal surface of the wall model. All FEA simulations were carried out using ANSYS Static Structural v19.2 (ANSYS, Canonsburg, PA, United States).

### 2.4. Haemodynamic metrics and endograft dynamics

This investigation explores the haemodynamic response to the implanted device, focusing on localised flow patterns, WSS-related metrics and flow disturbances. A list of the indices used here are defined in Table 2. A detailed description of the relevant haemodynamic metrics for the investigation can be found in our previous work (14).

**Table 2:** Haemodynamic indices used for analysis in this study.

Metric	Mathematical expression	Description
Time-averaged WSS	$TAWSS=\;\frac{1}{T}\int_{0}^{T}|\tau_{w}\;|dt$	Average of the WSS magnitude over the cardiac cycle.
Transverse WSS	$TransWSS\;=\;\frac{1}{T}\int_{0}^{T}\left|\tau_{w}\;.\;\left(n\;\times \;\frac{\int_{0}^{T}\tau_{w}dt}{\left|\int_{0}^{T}\tau_{w}dt\right|}\;\right)\right|dt$	Average over the cardiac cycle of WSS components perpendicular to the temporal mean WSS vector.
*λ*_2_ criterion	$\lambda_{2}=\;\frac{\partial v_{x}}{\partial y}\frac{\partial v_{y}}{\partial x}+{\left(\frac{\partial v_{y}}{\partial y}\right)}^{2}+\frac{\partial v_{y}}{\partial z}\frac{\partial v_{z}}{\partial y}$	Synthetic descriptor for incompressible flows used to evaluate isosurfaces in flow.
Displacement force	$\;F_{d,i}=\int_{A,i}\;pdA+\int_{A,i}\left(-\eta_{w}\frac{\partial u_{t}}{\partial \hat{n}}\right)dA$	Time dependent displacement force due to pressure and friction exerted by the flow of blood on the walls.
Oscillatory shear index	$OSI\;=\;\frac{1}{2}\left(1-\frac{\left|\int_{0}^{T}\tau_{w}dt\right|}{\int_{0}^{T}|\tau_{w}\;|dt}\right)$	Change of direction of the WSS vector from the primary direction of flow.
Endothelial cell activation potential	$ECAP\;=\;\frac{OSI}{TAWSS}$	Synthetic metric to identify regions at a higher risk of thrombus formation.

*T* is the time period of a cardiac cycle; *τ_w_* is the wall shear stress vector; *v_x_, v_y_, v_z_* are the velocity components in the *x, y,* and *z* direction; $u_{t}$ is the tangential velocity with respect to the unit normal for each element.

## 3. Results

### 3.1. Flow patterns

**Figure 7:**
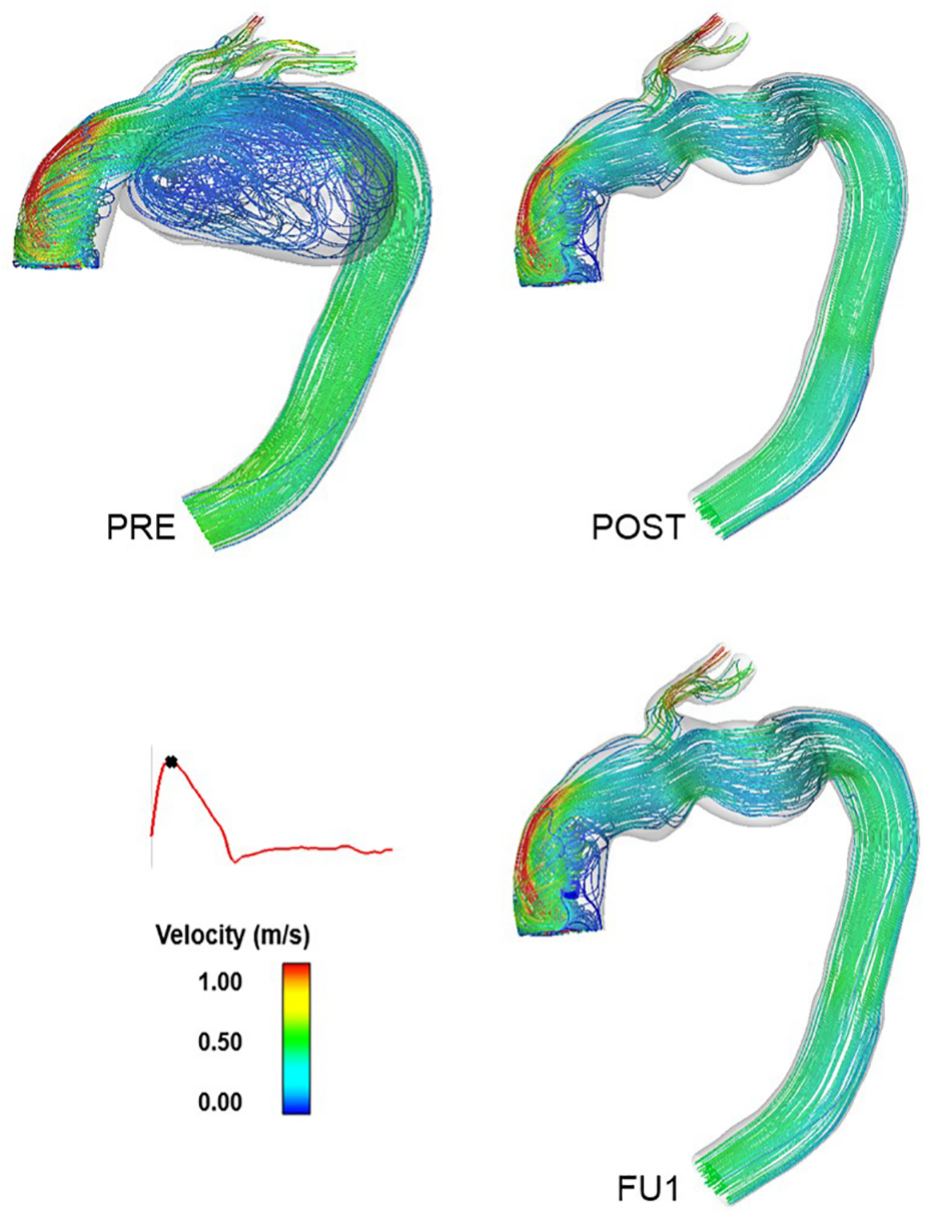
Instantaneous velocity streamlines at peak systole for the pre-TEVAR (PRE—top **left**) and post-TEVAR (POST—top **right**) and follow-up (FU1—bottom **right**) models. This indicates the exclusion of the aneurysm in the inner curvature of the arch following the procedure and the flow patterns observed at the follow-up examination following the compromise of the carotid-subclavian bypass.

Figure 7 shows instantaneous streamlines at peak systole for all the simulated scenarios (PRE, POST, and FU1). Flow in the ascending aorta was helical with high velocities skewed towards the outer curvature in all models. The presence of the aneurysm in the arch (PRE) caused a large recirculation zone in the aneurysm sac. Excluding the aneurysm *via* TEVAR restored a more desirable flow pattern in the arch, as can be seen in POST. This not only led to more uniform flow into the supra-aortic branches, but also virtually eliminated any undesirable recirculating flow in the arch. Aside from small differences in the supra-aortic branches, there seem to be no qualitative differences in flow patterns between POST and FU1.

**Figure 8:**
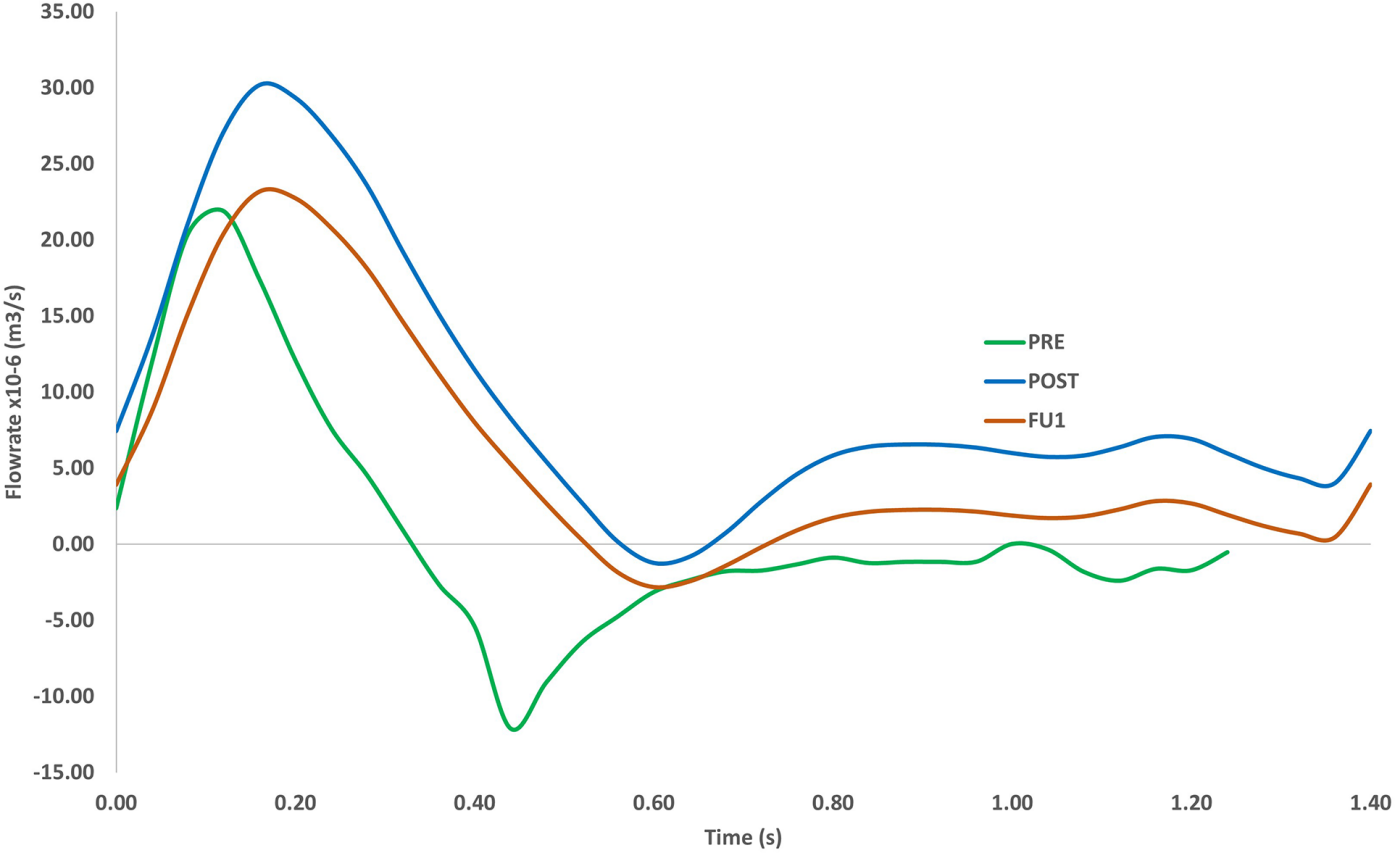
Volumetric flowrate in the RCC for the pre-TEVAR (PRE) case, post-TEVAR (POST) and follow-up (FU1) cases **(bottom)**. The length of the cardiac cycle was based on clinical measurements (1.23 s for pre-TEVAR and 1.4 s for post-TEVAR).

Comparison of the time-varying outflow through the RCC outlet is given in Figure 8 for the three simulated scenarios. The large recirculation zone in the aneurysm sac affected perfusion of the arch branches in PRE where flow through the RCC outlet was retrograde for approximately 64% of the cardiac cycle. PRE also had a lower peak flowrate and significantly lower flow throughout the cycle, providing a mean outflow of 3.48 × 10^−6 ^m^3^s^−1^ (0.21 L/min) through the RCC. Other arch branches also experienced large periods of retrograde flow ranging between 64%–69% of the cardiac cycle. Post-TEVAR flow through the RCC was mostly antegrade with a mean outflow of 9.59 × 10^−6 ^m^3^s^−1^ (0.57 L/min). The difference in outflow between POST and FU1 was due to the altered boundary conditions in FU1 to represent the break in the carotid-subclavian bypass further downstream of the RCC. As a result, FU1 had a lower flow rate of 5.55 × 10^−6 ^m^3^s^−1^ (0.33 L/min) through the RCC.

**Figure 9:**
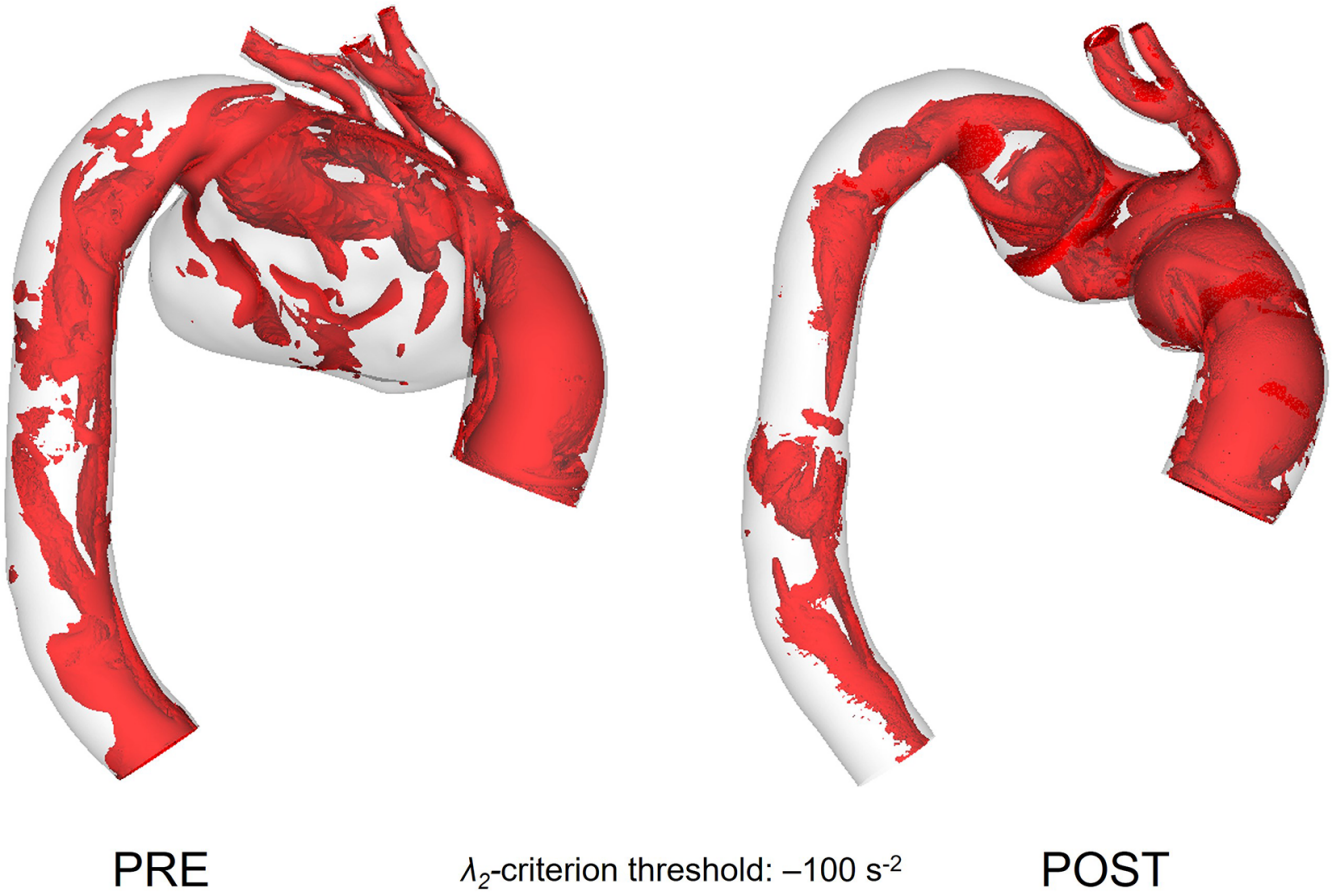
Vortical structures within the vessel represented as isosurfaces using the *λ*_2_ criterion for pre-TEVAR **(left)** and post-TEVAR **(right)** stages.

The vortical flow structure throughout the modelled aorta is visualised using the *λ*_2_ criterion as shown in Figure 9. A threshold value of −100 s^−2^ was chosen in order to isolate the relevant vortex cores formed in the aorta and to make suitable qualitative comparisons. Pre-TEVAR aneurysmatic flow understandably presented with a greater degree of disturbance as flow entered the arch branches compared to the post-TEVAR model which presented with vortical flow that can be often seen in and attributed to the curved nature of the vessel giving rise to counter-rotating vortices.

### 3.2. Wall shear stress related indices

**Figure 10:**
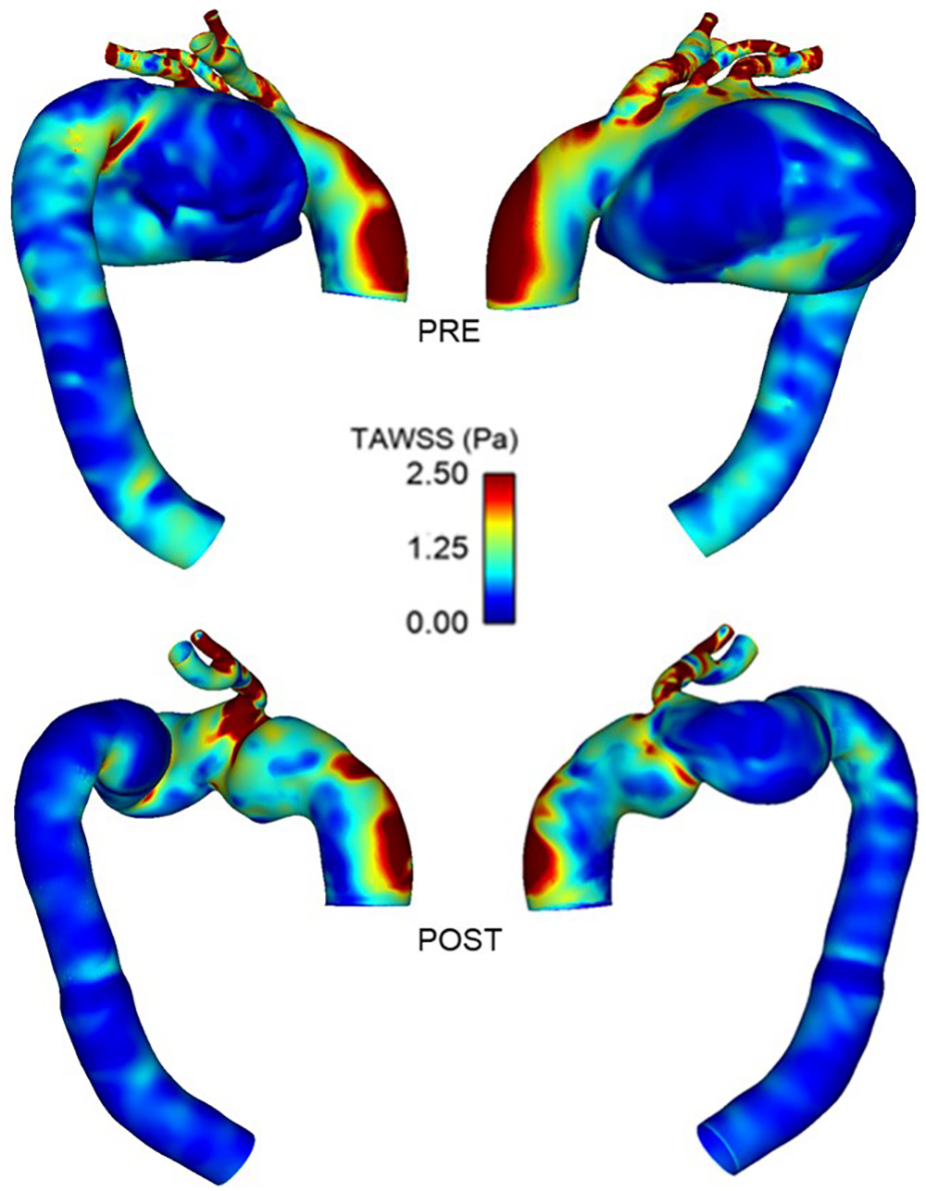
Comparison of time-averaged WSS between pre-TEVAR **(top)** and post-TEVAR **(bottom)** stages.

Wall shear stress and its associated indices are important near wall haemodynamic parameters which can affect endothelial cell proliferation and play a role in thrombus formation (35, 36). Figure 10 demonstrates the difference in time-averaged WSS (TAWSS) patterns between PRE and POST. Both present with a large area of high TAWSS along the outer curvature of the ascending aorta, which is due to the skewed inlet velocity profile and the curvature of the aorta. The large recirculation zone in the aneurysmal sac, as seen in Figure 7, led to extremely low TAWSS in this area (PRE), with elevated TAWSS along the outer curvature of the arch and in the emerging branches. In the PRE model, a patch of elevated (>2.5 Pa) TAWSS can be seen in the inner curvature of the arch, immediately downstream of the aneurysm.

**Figure 11:**
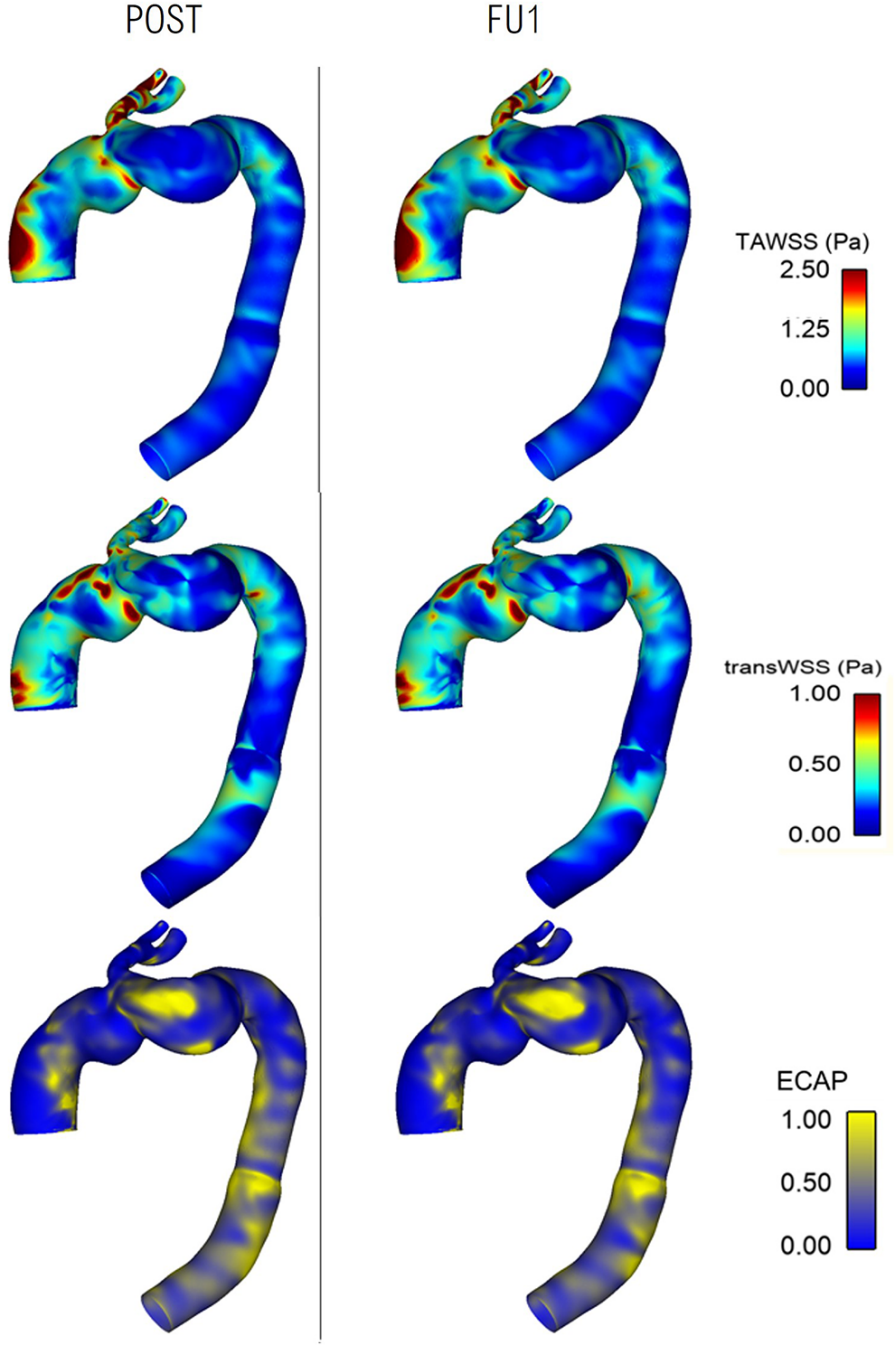
Comparison of wall shear stress-related metrics between post-TEVAR and follow-up stages with time-averaged WSS **(top)**, transverse WSS **(middle)** and endothelial cell activation potential **(bottom)** maps all showing similar spatial distribution and patterns between the two cases.

Comparisons of WSS-related indices between POST and FU1 are shown in Figure 11, displaying high degree of similarities. TAWSS and transverse WSS (transWSS) are useful metrics which serve as indicators for thrombus formation and plaque development, and there appears to be no extremes of magnitudes or abnormal spatial distribution of either. Endothelial cell activation potential (ECAP) identifies regions of high oscillatory shear index (OSI) and low TAWSS and regions of ECAP higher than 5.0 can pose a risk of thrombus formation (37, 38). However, in these cases there appears to be no abnormally high values of ECAP.

### 3.3. Displacement force

**Figure 12:**
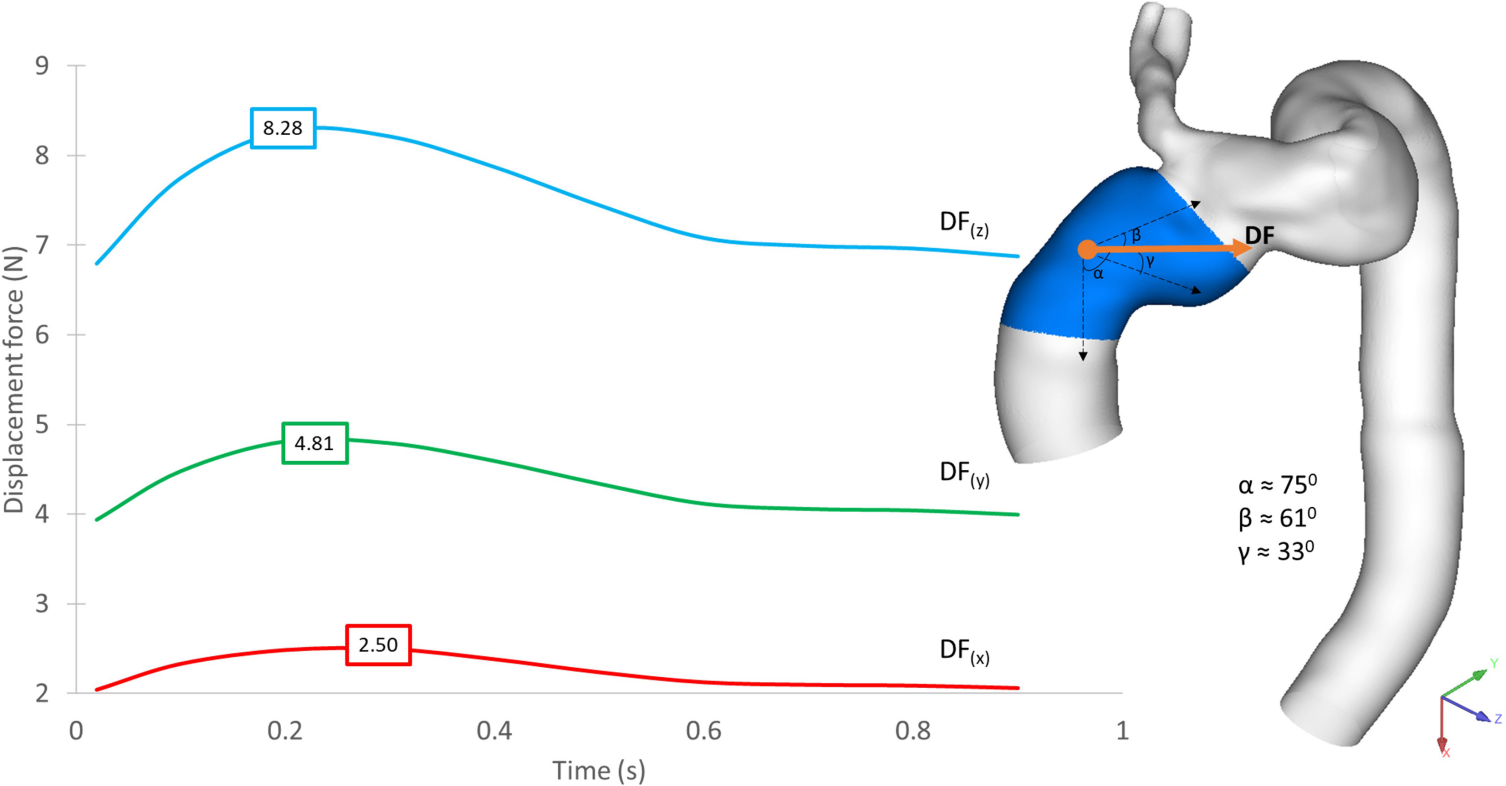
Time-dependent displacement forces acting on the ascending module of the device, shown for POST, decomposed into x-, y- and z-components with peak values in each direction indicated on the plot. The direction of the resultant force is shown on the geometric model.

The implanted SG experiences a displacement force (DF) resulting from the change in net momentum owing to pressure and WSS generated by the aortic blood flow. Since pressure is the dominant component, the time-varying nature of displacement force is expected to closely resemble the pressure waveform (39). Figure 12 shows the displacement force acting on the ascending module of the endograft in POST, decomposed into three orthogonal components as defined in the figure. The displacement force was primarily in the z-direction but with a significant component in the y-direction due to the non-planar curvature of the aorta, thus along the coronal plane for the individual.

**Figure 13:**
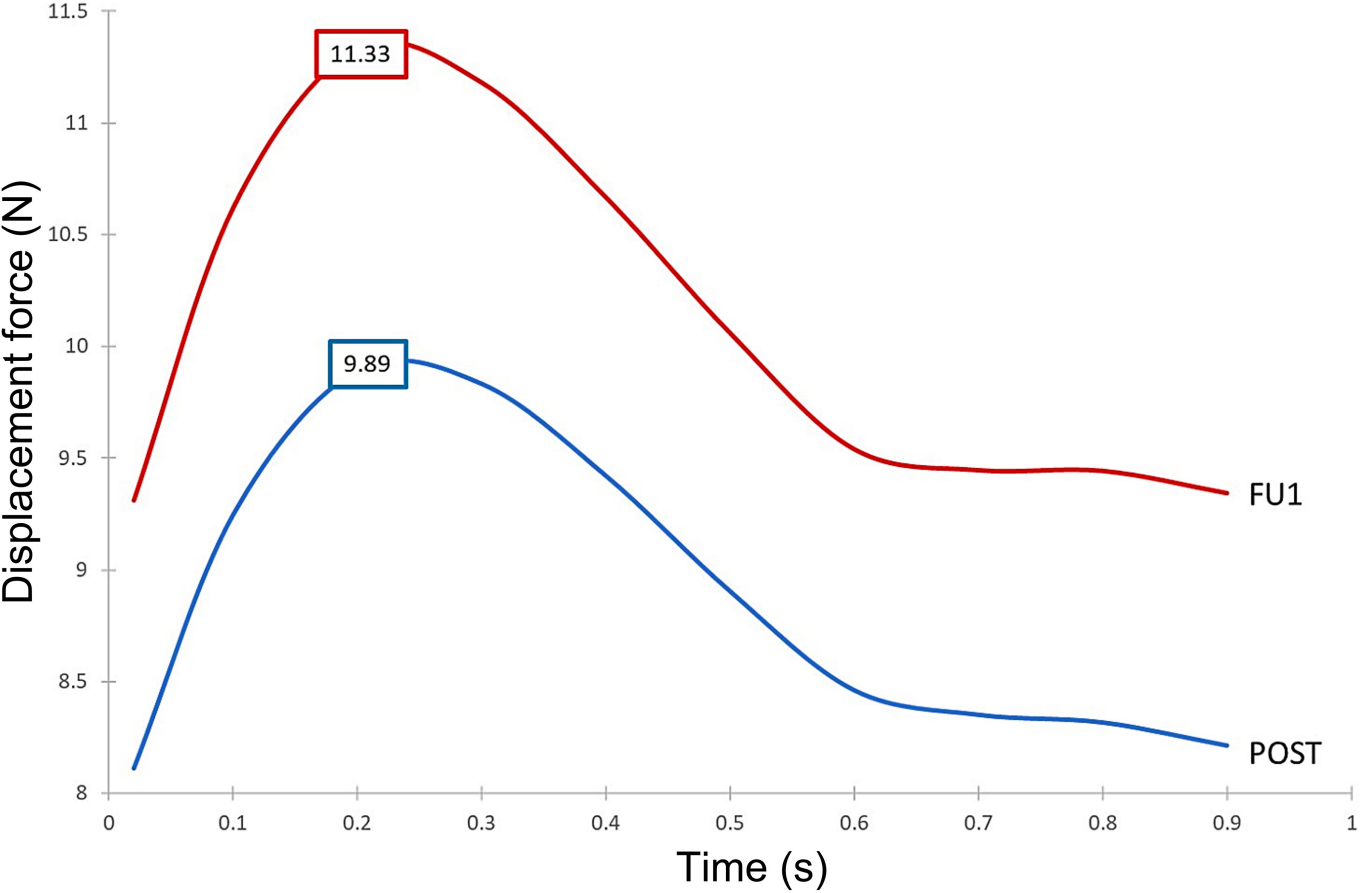
Time-dependent displacement forces exerted on the ascending module of the device post-TEVAR (post) and at follow-up (FU1) stages.

Figure 13 demonstrates the difference in displacement force between POST and FU1, with the endograft experiencing a consistently higher force in FU1 than in POST throughout the cycle. The maximum displacement force in FU1 was 15% higher than in POST.

**Figure 14:**
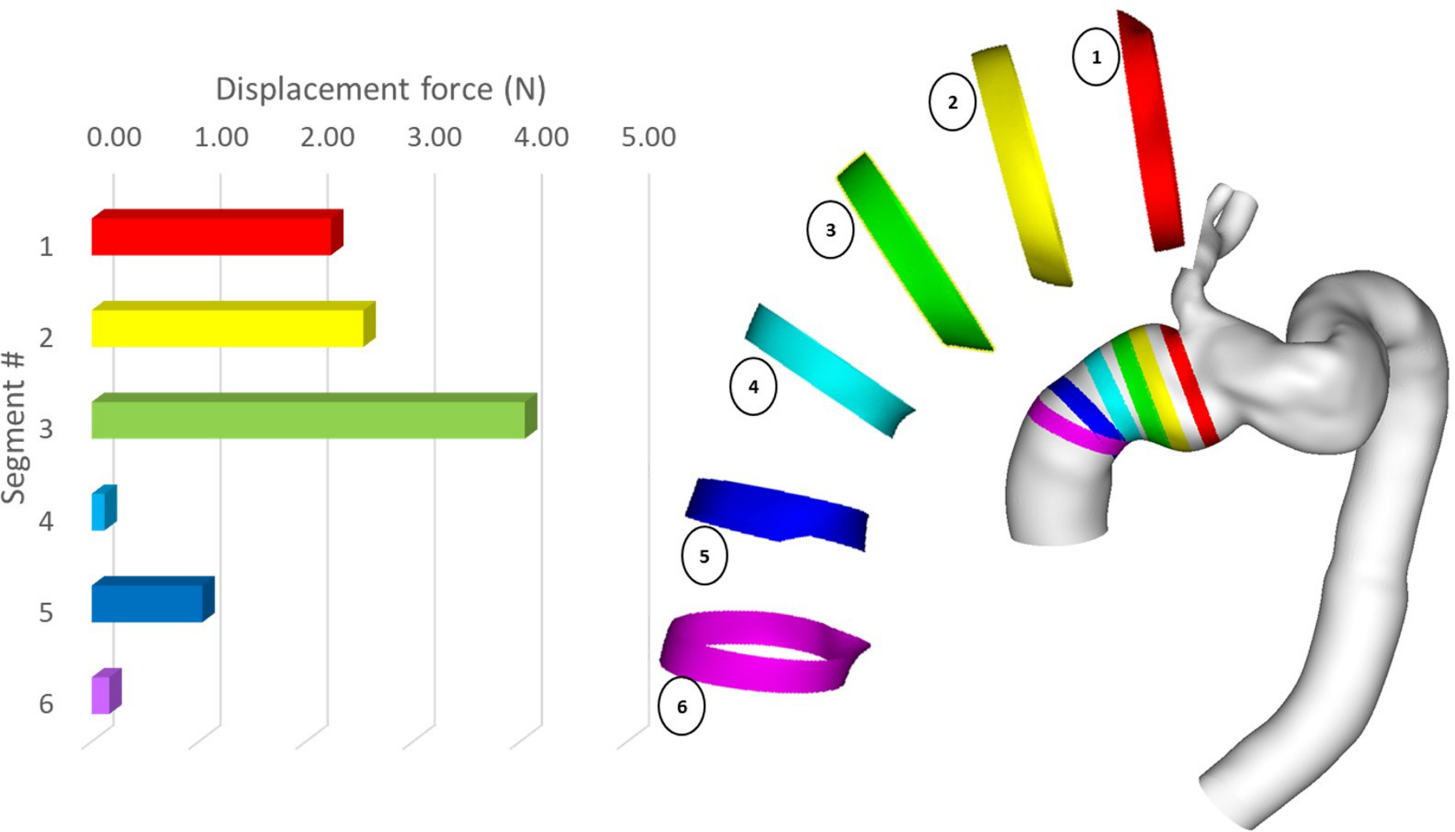
Peak magnitude of displacement force exerted upon different segments of the ascending module of the device.

Since the ascending module of the device is of particular interest in this case, it was further divided into separate segments, and the magnitude of displacement force was calculated for each of these segments. As shown in Figure 14, the distribution of displacement force was non-uniform with larger values in the distal portion of the ascending module. Segment 3 experienced the largest displacement force, followed by segment 2 and 1, which are closer to the overlapping region between the ascending module and the main endograft.

### 3.4. Wall displacement and mechanical stress

**Figure 15:**
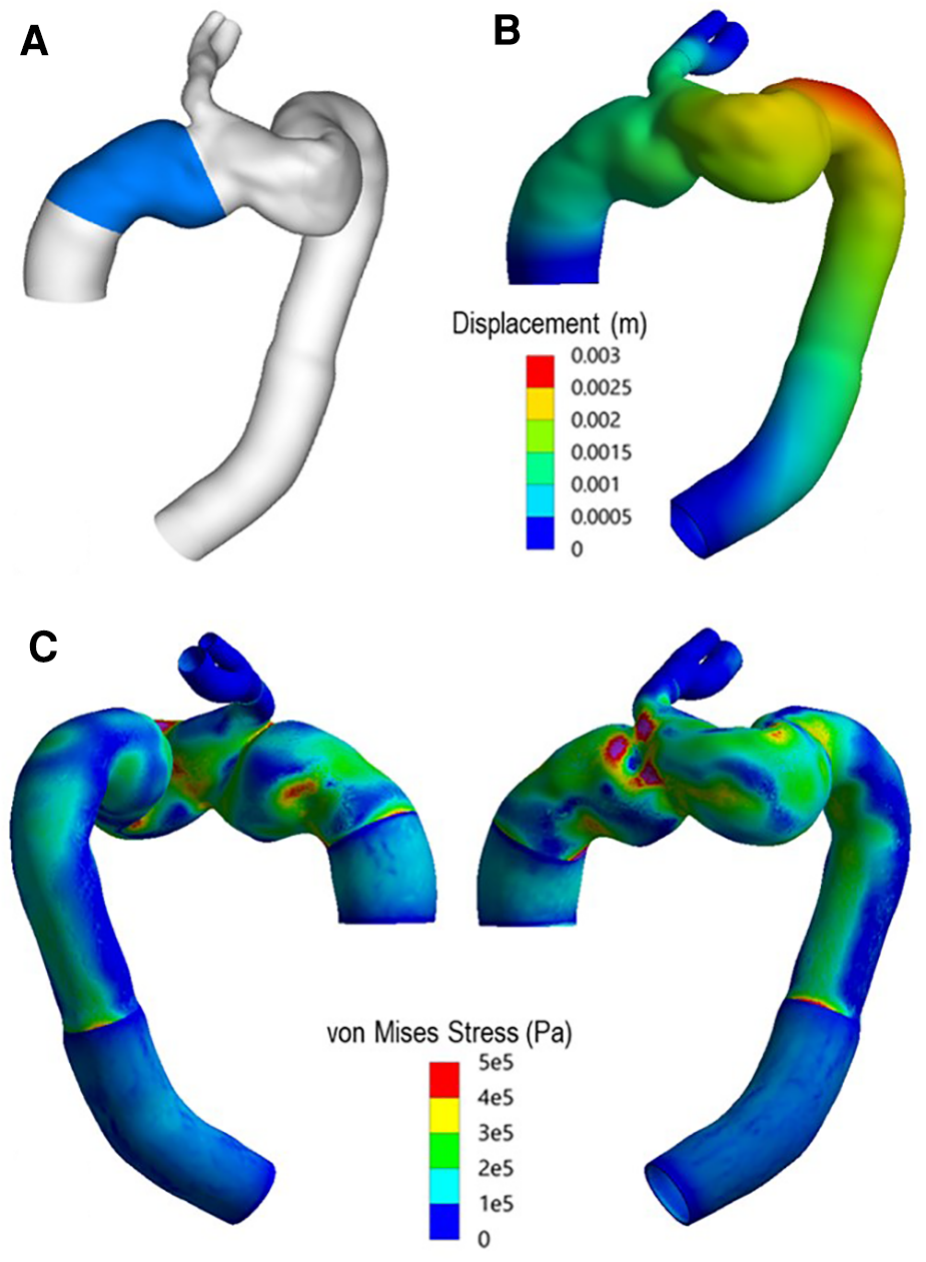
(**A**) Highlighted region indicates ascending module of the device. (**B**) Total displacement experienced by the vessel at peak systole. (**C**) Spatial distribution of von Mises (equivalent) stress in the wall at peak systole.

Figure 15 presents the spatial distribution of wall displacement and von Mises stress (equivalent stress) obtained with the FE analysis. The structural analysis was carried out at peak systole to represent the worst-case scenario when the aorta was subjected to the maximum pressure load. The highest von Mises stresses were observed in the distal ascending aorta, immediately upstream of the emerging branch. This coincides with the previously highlighted overlapping region between the two modules. The maximum displacement of up to 2.41 mm was observed at the distal end of the arch and could be attributed to the region not being anchored by the LCC and LSA.

## 4. Discussion

The case analysed in this investigation presented with a rather complex pathology along with several comorbidities that had to be taken into account when determining treatment for the patient. TEVAR was considered the best option due to its minimally invasive nature and the availability of the device to exclude the aneurysm and restore flow in the region. The chosen device was a single-branched aortic arch endograft with a side branch leading into the innominate artery. Revascularisation was carried out prior to the TEVAR procedure by introducing bypasses from the RCC to LCC and LCC to LSA. Clinical information and the second follow-up CT examination (as shown in Figure 2 at FU2) indicated the formation of a new aneurysm in the ascending aorta, with migration of the ascending module of the device and a suspected leak or tear in the ascending aorta.

### 4.1. Comparing pre- and post-intervention haemodynamics

As shown in Figure 7, the presence of a large aneurysm in the arch caused a significant reduction in flow velocity and recirculating flow in this region, which adversely influenced the flow leading into the arch vessels as reported by others (13). This was evidenced through a prolonged period of retrograde flow through the RCC (Figure 8), which could impair blood perfusion further downstream. Multidirectional flow can also lead to extreme WSS, which may increase the risk for thrombus formation and/or atherosclerotic lesion development in the supra-aortic arteries (40).

The TEVAR procedure using a single branched endograft successfully excluded the aneurysm and restored more organised flow in the arch. A smoother lumen, in the absence of the aneurysm, allowed for sufficient flow leading into the IA, which in turn perfused the LCC and LSA through the bypass performed pre-TEVAR. Nevertheless, the non-planar and tortuous geometry of the arch was exacerbated after the endograft was deployed as can be seen from the post-TEVAR geometry (Figure 4). The procedure also resulted in increased flow into the IA, which was the only supra-aortic branch directly perfused through the arch and it had to carry additional flow to supply the LCC and LSA. The choice of device was made based on the complex nature of the region being treated and to ensure sufficient perfusion to the supra-aortic branches. Branched and fenestrated endografts would both serve the purpose of aortic branch perfusion, but the local haemodynamics will be influenced by the endograft design. Using a branched stent-graft allows for flow to be smoothly guided into the emerging arch branch. Additionally, the ability of the main module of the single branched device to be securely anchored in the deployed region made it the more suitable choice in this case.

### 4.2. Post-intervention and beyond

Flow patterns in the POST and FU1 were largely similar throughout the aorta, with a small difference in the IA due to the different outflow through the RCC branch. Close examination of WSS-related indices also revealed no significant alteration between POST and FU1 as shown in Figure 11, suggesting that the minor change in outflow conditions through the RCC in FU1 had not affected the global flow patterns and near wall haemodynamics.

It was clear from the final follow-up (FU2) CT scan (Figure 2) that further complications occurred between FU1 and FU2. The scan revealed the formation of a large aneurysm alongside the ascending module of the device, which extended along the outer curvature of the arch. Flow through this bulge perfused the native ostia of the LSA and LCC which were initially occluded prior to the TEVAR procedure. It was suspected that a leak or tear occurred in the ascending aorta, but the origin of the leak was undetermined, and a suspected source of the leak could be migration of the device or dehiscence due to improper fixation. This necessitated a closer examination of the biomechanical environment of the ascending aorta in searching for a plausible cause for the suspected leak.

Firstly, the possibility of device migration was assessed. The main module of the device was unlikely to move as it was anchored securely by the branch leading into the IA, but the ascending module was connected to the main module through a self-protecting sleeve and relied on a radial force interlocking mechanism to hold it in place. Migration of the ascending module could occur if the displacement force (DF) acting on it was sufficient to move it upward or pull it away from the main module, which would lead to type I or type III endoleak respectively (41). Previous studies suggested that DF exerted on the endograft can have a considerable effect on its spatial stability (42), and that the magnitude and direction of DF are influenced by the endograft geometry, the haemodynamic state of the patient, and the local geometry of the vessel (39, 43–45). Therefore, it was necessary to evaluate the DF experienced by the ascending module in both POST and FU1.

Our results showed that despite overall flow patterns being largely similar between POST and FU1, the DF increased by approximately 15% in FU1. The only difference between the POST and FU1 models was the outflow conditions imposed at the outlets. As has already been mentioned, the boundary conditions were altered to reflect the compromised LCC-LSA bypass, with excess flow being diverted to the descending aorta. This redirected flow resulted in changes in the normal pressure forces on the vessel wall, leading to an increase in DF. Nevertheless, the maximum DF of 11.33 N in FU1 was well below the reported threshold of 32 N to dislocate a non-planar stent-graft in the thoracic aorta (46). While threshold values have been reported for the abdominal and thoracic aorta (47, 48), there is little information in the literature on the magnitude of DF needed to cause device migration in the ascending aorta. It was also interesting to note the direction of DF as illustrated in Figure 12 which shows clearly that the total DF experienced by the ascending module deviates from its local longitudinal direction. This was attributed to the curvature and non-planarity of the aorta, especially in the region of interest here, giving rise to increased anterio-posterior and lateral components (43). The direction of DF vector indicated that it would pull the ascending module laterally away from the outer curvature, which could compromise the stability in the proximal landing zone or lead to misalignment in the device (18, 19). Further analysis of the distribution of DF along the ascending module showed that the region close to the connection between the two modules experienced relatively high DF (Figure 14).

The spatial distribution of von Mises stress obtained with the finite element analysis showed high stresses in the region where the two parts of the device connect (Figure 15), owing to the highly tortuous local geometry and the emergence of the IA branch. The maximum von Mises stress was approximately 1.3 MPa, which exceeded the yield stress for dilated ascending aorta of 1.2 ± 0.1 MPa referenced in other studies (49, 50). Although the maximum stress occurred in the wall protected by the endograft, and the graft is much stiffer and can withstand higher stresses compared to the native aorta, the extremely high level of stress in this region could compromise the device locking mechanism, thereby increasing the risk of disconnection between the two modules. Moreover, high stress concentrations were also observed in the proximal and distal ends of the device, resulting from local geometric discontinuity and compliance mismatch between the graft and the native aorta. Such focal high stress regions have been found to correlate strongly with the locations of stent-graft induced new entry in type B aortic dissections (51, 52). Based on these findings, it is plausible to speculate that a proximal tear might have occurred which then led to the formation of the observed aneurysm.

### 4.3. Limitations

In the CFD simulations presented in this investigation, the aortic wall was assumed to be rigid and the supra-aortic vessels bypass was not included in the post-TEVAR model. The rigid wall assumption is expected to have a minor influence on the predicted flow patterns, especially in the post-TEVAR and follow-up models where a large part of the aorta was supported by the endograft. Excluding the supra-aortic vessels bypass is also likely to have an insignificant effect on the predicted haemodynamics and wall stress, even though its inclusion would have provided the opportunity to investigate potential causes for the failed carotid-subclavian bypass observed at FU1. In the finite element stress analysis, the aortic wall was modelled as a linear elastic material; this assumption was made because within the region of interest, the post-TEVAR aortic wall was largely integrated with the endograft with a relatively small section of the native aorta at the proximal and distal end of the device. In addition, the periodic motion of the aortic root and its influence on the ascending aorta were ignore, which could have influenced the predicted wall stress (53). Finally, the results presented here were confined to a single patient. However, the number of patients undergoing TEVAR of the entire aortic arch with a single-branched device is very limited and this paper focused on presenting a longitudinal analysis at multiple stages of the treatment.

## 5. Conclusion

This investigation presents a detailed haemodynamic and biomechanical analysis of a patient who underwent TEVAR treatment for a large aneurysm in the aortic arch using a single-branched endograft. Imaging data from different stages of the process along with physiologically representative numerical modelling allowed to paint a picture of the progression of the case from pre-intervention to post-intervention and beyond. Simulation results for the different stages demonstrated the dramatic improvement in flow patterns in the aortic arch after the TEVAR procedure. As the final follow-up examination revealed the formation of a new aneurysm in the ascending aorta, further analysis was carried out in searching for possible causes for the observed complication. Results for displacement forces on the endograft and stresses within the wall indicated that the endograft was subjected to an angulated displacement force in the lateral direction and the overlapping region between the main and ascending module experienced very high stresses, which could act together to weaken the locking system, resulting in migration or misalignment of the device. In addition, high stress concentration was observed at the proximal end of the ascending module, suggesting the possibility of a proximal tear as a source for the observed aneurysm. Careful positioning of the overlapping region and the proximal landing zone may help reduce the stresses in these regions at risk of compromising stent-graft stability. In this regard, finite element-based simulations of virtual stent-graft deployment, such as those reported recently on aortic dissections (53, 54), offer a promising tool that should be further developed and validated for use in pre-intervention planning to minimise potential device migration or endoleaks.

## Data Availability

The raw data supporting the conclusions of this article will be made available by the authors, without undue reservation.
